# The effects of patient cost-sharing on health expenditure and health among older people: Heterogeneity across income groups

**DOI:** 10.1007/s10198-021-01399-6

**Published:** 2021-11-15

**Authors:** Hirotaka Kato, Rei Goto, Taishi Tsuji, Katsunori Kondo

**Affiliations:** 1 Graduate School of Business Administration, Keio University, 4-1-1 Hiyoshi, Yokohama, Kanagawa 223-8521 Japan; 2 Center for Preventive Medical Sciences, Chiba University, 1-8-1 Inohana, Chuo-ku, Chiba, 260-8670 Japan; 3 Center for Gerontology and Social Science, National Center for Geriatrics and Gerontology, 7-430 Morioka-cho, Obu-shi, Aichi, 474-8511 Japan; 4 Graduate School of Health Management, Keio University, 35 Shinanomachi, Shinjuku-ku, Tokyo, 160-8582 Japan; 5 Faculty of Health and Sport Sciences, University of Tsukuba, 3-29-1 Otsuka, Bunkyo-ku, Tokyo, 112-0012 Japan

**Keywords:** Health expenditure, Cost-sharing, Older people, Income inequality, Regression discontinuity design, I13, I14, I18

## Abstract

Despite rapidly rising health expenditure associated with population aging, empirical evidence on the effects of cost-sharing on older people is still limited. This study estimated the effects of cost-sharing on the utilization of healthcare and health among older people, the most intensive users of healthcare. We employed a regression discontinuity design by exploiting a drastic reduction in the coinsurance rate from 30 to 10% at age 70 in Japan. We used large administrative claims data as well as income information at the individual level provided by a municipality. Using the claims data with 1,420,252 person-month observations for health expenditure, we found that reduced cost-sharing modestly increased outpatient expenditure, with an implied price elasticity of  – 0.07. When examining the effects of reduced cost-sharing by income, we found that the price elasticities for outpatient expenditure were almost zero,  – 0.08, and  – 0.11 for lower-, middle-, and higher-income individuals, respectively, suggesting that lower-income individuals do not have more elastic demand for outpatient care compared with other income groups. Using large-scale mail survey data with 3404 observations for self-reported health, we found that the cost-sharing reduction significantly improved self-reported health only among lower-income individuals, but drawing clear conclusions about health outcomes is difficult because of a lack of strong graphical evidence to support health improvement. Our results suggest that varying cost-sharing by income for older people (i.e., smaller cost-sharing for lower-income individuals and larger cost-sharing for higher-income individuals) may reduce health expenditure without compromising health.

**Supplementary Information:**

The online version contains supplementary material available at 10.1007/s10198-021-01399-6.

## Introduction

Rapidly rising health expenditure associated with population aging is a major concern in many developed countries. Greater cost-sharing plays an important role in mitigating the moral hazard problem involved in healthcare insurance, thus containing health expenditure. Cost-sharing can, however, also be a strong barrier to receiving necessary healthcare services, which raises serious concerns about adverse health effects, especially for low-income individuals. In fact, a large body of literature shows that low income is associated with poor health [1–5]. These health inequalities between the rich and poor might, at least partially, result from the under-use of health care because user fees play a more important role in utilization decisions among low-income individuals [6, 7]. To implement efficient and equitable health insurance policies targeting older people, understanding not only the effects of cost-sharing on the overall population but also the heterogeneous effects of cost-sharing by income is essential.

There is a small but growing body of literature on the effect of cost-sharing on older people [8–10], but the existing studies focused only on a small set of services or on relatively wealthy older people. Therefore, their findings may not be generalizable to a broader range of services or to people with middle and low incomes. More importantly, there is little evidence as to the heterogeneous effects of cost-sharing by income on older people. Even for other age groups, very few studies have examined heterogeneous responses by income in a credible way [11–13]. Thus, it remains unclear whether the effects of cost-sharing on the utilization of healthcare and health vary by income.

To bridge this gap in the literature, the present study empirically examined the effects of cost-sharing on the utilization of healthcare and health, using data from older people in Japan. We also examined the heterogeneous effects of cost-sharing by income. Japan’s health insurance system provided a unique opportunity to assess the effect of cost-sharing on older people. Older people in Japan experienced a drastic reduction in the coinsurance rate from 30 to 10% at age 70, allowing us to robustly estimate the effect of cost-sharing. As this reduction in coinsurance applied to older people within a wide range of income levels, we can estimate the overall effects of cost-sharing but also separate effects for lower-, middle-, and higher-income individuals in our sample.

We relied on a sharp regression discontinuity (RD) design to identify the impact of cost-sharing on the utilization of healthcare and health, taking advantage of the reduction in the coinsurance rate from 30 to 10% at age 70. Using ordinary least squares (OLS), we examined the overall effects of cost-sharing and the heterogenous effects by income. To examine the heterogeneous effects, we conducted stratified analyses by income. We used a large individual-level claims dataset from September 2011 to March 2014 and income-related insurance premium information provided by a municipality in Japan. This municipality is a typical city in Japan in terms of the proportion of older people [14] and the distribution of income-related insurance premium groups [15]. These data allow us to accurately capture health expenditure as well as income at the individual level. In order to assess health effects, we also used mail survey data from a random sample of older people in the municipality.

Our key findings are as follows: First, when examining the overall sample of older people, we found that the reduction of coinsurance rate from 30 to 10% increased the expenditure on outpatient care by 4.8%. The implied price elasticity for outpatient expenditure was  – 0.07. For inpatient care, we did not find any evidence that reduced cost-sharing changed the utilization of care. Second, when examining the effect of reduced cost-sharing on outpatient expenditure by income, we found no evidence that reduced cost-sharing changed the utilization of outpatient care for lower-income individuals, but we found a 5.4% increase for middle-income individuals and a 7.7% increase for higher-income individuals. The implied price elasticities for outpatient expenditure were almost zero,  – 0.08, and  – 0.11 for lower-, middle-, and higher-income individuals, respectively, suggesting that lower-income individuals do not have a more elastic demand for outpatient care compared with other income groups. These findings may be surprising given that there is a lot of speculation that low-income groups have more elastic demand [11]. In the Discussion section, we consider potential reasons for why lower-income individuals had less elastic demand than other income groups. Finally, we found that cost-sharing reduction significantly improved self-reported health only among lower-income individuals, but it was hard to draw clear conclusions about health outcomes because of the lack of strong graphical evidence to support the health improvement.

The remainder of this paper proceeds as follows: In Sect. “Literature review”, we review previous studies on this topic. Section 3 provides background information on the institutional setting. Sections 4 and 5 present the data and estimation strategy used in this study. Section 6 presents our results, and in Sect. 7 we discuss our results.

## Literature review

One of the most credible studies on the effects of cost-sharing is the RAND Health Insurance Experiment (HIE), a randomized controlled trial conducted in the 1970s [16]. It found that the average price elasticity of demand for healthcare services was around  – 0.2 across different types. Additionally, on average, there was no evidence of an adverse health impact arising from greater cost-sharing in the experiment.

An important goal of the RAND HIE was to examine how the response to cost-sharing varies by subgroup. The experiment found greater effects on the low-income and sicker groups. Regarding health outcomes, there were nontrivial changes due to cost-sharing among chronically ill low-income groups. However, the findings of the RAND HIE may not be applicable to older people because the RAND HIE did not include older people. In addition, given that there have been significant improvements in medical practices since the experiment, which may have led to a structural change in the elasticity of medical demand and health impacts of cost-sharing, the results of the experiment may not be directly applicable to today’s scenario.

Since the RAND HIE, numerous studies have exploited a policy as a natural experiment to investigate the effects of cost-sharing on adults [17, 18], children [13, 19, 20], and older people [8–10]. Chandra et al. [8] examined the substitution between outpatient and inpatient care among older people in the U.S.—whether increases in cost-sharing for prescription drugs and physician visits affect hospital utilization. In contrast, we examined the effects of an across-the-board reduction in cost-sharing on health expenditure.

Our study is closely related to that of Shigeoka [9] and Fukushima et al. [10] who exploited the same cost-sharing reduction at age 70 in Japan, which was also examined in our study. The most important difference between our study and these two works is the data used. Shigeoka [9] used survey data that do not contain information on health expenditure and cover only limited types of services. To complement the study of Shigeoka [9], Fukushima et al. [10] used claims data that contain information on all services provided and associated health expenditure. However, Fukushima et al. [10] focused on relatively wealthy older people because their claims data came from employee-based public health insurance managed by Health Insurance Societies, which mainly covers large companies. Most older people—75% of older people aged 65–74 in 2014—are enrolled in community-based public health insurance instead of employee-based public insurance, and the average income of enrollees for community-based public insurance is less than half that of enrollees for the society-managed plan[21].^1^ In the present study, we used claims data from community-based public health insurance in a typical municipality with respect to the distribution of income-related insurance premium groups.

The evidence on whether the effects of cost-sharing vary by income is extremely limited [11–13]. Several studies have explored the heterogeneous effect of cost-sharing by income [22–27]. However, income was poorly measured in these studies, which used self-assessed income or regional income as a proxy for individual income. Additionally, almost all studies did not examine the heterogeneous effects on health. Several studies examined the effect of cost-sharing among low-income individuals [17, 28], but because these studies focused on the programs targeted at only low-income individuals, these findings are not directly comparable with estimates for high-income individuals obtained from other contexts.

Only three recent studies [13, 18, 20] using a quasi-experimental design examined the heterogeneous effects of cost-sharing in a creditable way. They used income at the individual level and compared the effects of cost-sharing on the utilization of care across different income groups among children and young adults in Sweden and Taiwan. Unlike these three studies, the present study focused on older people, the most intensive users of health care. Additionally, these three studies did not examine the heterogeneous effects by income on health. Taken together, the present study is the first to examine the heterogeneous effects of cost-sharing on both healthcare utilization and health by income among older people using income information at the individual level.

## Institutional background

All residents in Japan are mandatorily covered by public health insurance. Employees and their dependents are enrolled in employee-based public insurance, and those not covered by employee-based public insurance are enrolled in community-based public insurance unless they are on public assistance.^2^ The benefit packages and fee schedules are uniformly set by the government, regardless of insurance type. The benefit packages are comprehensive, including inpatient and outpatient services, prescription drugs, and basic dental services. Thus, the composition of insurance enrollees would not be endogenously determined by the level of cost-sharing and benefit packages in our case, which often complicates U.S. studies.

There is no gatekeeping system in Japan. Patients can visit any provider, including specialists and teaching hospitals, without a referral, although additional fees may be required for a hospital visit without a referral.

Cost-sharing in Japan takes the form of coinsurance, which is the percentage of healthcare costs that patients incur. There is no deductible amount in Japan, in contrast to normal health plans in the U.S. Patients pay coinsurance at the provider’s office when they visit the provider, and the insurers reimburse the rest. The same coinsurance rate applies to all medical services, including inpatient, outpatient, and prescription drugs. To protect patients against catastrophic health expenditure, cost-sharing is reduced when the monthly out-of-pocket payment exceeds a threshold value (for more details, refer to Supplementary Appendix A).

Cost-sharing depends on income and age in Japan. In addition to the national cost-sharing schedule, some local governments provide their own subsidies. Table 1 summarizes the cost-sharing schedule in the municipality of our study. The insurance premium category shows an individual’s income level. As shown in Table 1, older people in categories 3–8 experience a drastic coinsurance rate reduction from 30 to 10%, a decline of 67%, in the next month after turning age 70.

**Table 1:** Cost-sharing policy in the examined municipality during our study period (September 2011 to March 2014)

Insurance premium category	Definition in our study	Explanation	Ratio of each category population, %	Below 70		Between 70 and 74	
				Coinsurance, %	Stop-loss: maximum out-of-pocket payment per month, JPY	Coinsurance, %	Stop-loss: maximum out-of-pocket payment per month, JPY
1		The individual on public assistance	5.0	0		0	
2		All family members, including the individual, are exempted from the municipal tax, and the individual’s total amount of income is < = 800,000 JPY	20.3	10 or 20 (depending on income)	15,000 or 35,400 (depending on income)	10	15,000
3, 4	Lower-income individuals	All family members, including the individual, are exempted from the municipal tax, and the individual’s total amount of income is > 800,000 JPY	13.7	30	35,400	10	24,600
5, 6	Middle-income individuals	At least one family member is not exempted from the municipal tax, but the individual is exempted from the municipal tax	23.8	30	80,100 + (medical costs − 267,000) × 1%	10	44,400
7, 8	Higher-income individuals	The individual is not exempted from the municipal tax, and the individual’s total amount of income is < 2,000,000 JPY	21.4	30	80,100 + (medical costs − 267,000) × 1%	10	44,400
9, 10, 11, 12		The individual is not exempted from the municipal tax, and the individual’s total amount of income is = > 2,000,000 JPY	15.8	30	150,000 + (medical costs − 500,000) × 1%	30	80,100 + (medical costs − 267,000) × 1%

Note: The insurance premium category is the category for the public long-term care insurance in the municipality. Individuals in category 2 are subsidized by the local government. Although lower-, middle-, and higher-income individuals experience the cost-sharing policy explained in Table 1, some individuals in the other category experience a different cost-sharing policy because the insurance premium category does not completely coincide with that policy. For example, depending on income, the cost-sharing policy for some individuals in category 9 is the same as that for high-income individuals. Medical costs stand for the total medical cost, including both the patient’s out-of-pocket payments and the amount that the insurer reimburses to the provider. Additional details on the cost-sharing rule are not described in this table. First, if patients are eligible for the stop-loss more than three times within a 12-month period, the stop-loss of the patient is further enhanced. Second, for individuals aged between 70 and 74 years, an additional stop-loss of 12,000 JPY per month applies separately to outpatient services. We took these details into account when calculating cost-sharing. However, we acknowledge that we could not take some rules into account. For example, although the stop-loss should apply to the household instead of individually, we could not do this because we do not have information on family relationships

If turning age 70 coincides with changes in any other factors such as employment or receiving a pension, which affect the demand for healthcare, it is impossible to isolate the effect of cost-sharing. Therefore, it is important to understand whether there are other changes at age 70. First, there is little concern about the change in employment status because the mandatory retirement age in almost all firms in Japan is age 60 or 65. Second, there is little concern about receiving pensions, as public pension payments start at age 60, not at age 70. In fact, as shown later, we did not find any evidence that there is a discontinuity in income at age 70, although we would expect income to change at 70 if the changes in employment status or receiving a pension coincided with turning age 70. Third, there is no change in the provider’s incentives at age 70 because providers receive the same payments regardless of patients’ age as long as they provide the same treatment.

## Data

In the present study, we used three types of data from a municipality in Japan: administrative claims data on community-based public health insurance, enrollment data, and income data (insurance premium category data). The municipality is a typical city in Japan with respect to the proportion of older people [14] and the distribution of income-related insurance premium groups [15].

Our claims data cover outpatient care (including prescription drugs) and inpatient care from September 2011 to March 2014. From the claims data, we measured the healthcare expenditure of each person per month. From the enrollment data, we observed the enrollee’s birthday and gender along with the period in which they were enrolled.^3^ We focused on individuals aged between 68 and 71 years. If a person was admitted into or withdrew from community-based public health insurance, we excluded that person’s claims data for the month of admission/withdrawal because we were unable to measure full health expenditure for that month.

For income information, we obtained the insurance premium category of each person for the public long-term care insurance determined by the municipality. All individuals aged 65 years and above in the municipality were classified into 12 categories based on their income and that of other household members in 2012.^4^ Table 1 reports the cost-sharing policy of each category. In this study, we focused on categories 3–8 because individuals in the other categories did not experience cost-sharing reduction. As a result, our main data did not include very low-income individuals (the bottom 25% of the income distribution) or very high-income individuals (the top 16% of the income distribution). We defined individuals in categories 3 and 4 as lower-income individuals, 5 and 6 as middle-income individuals, and 7 and 8 as higher-income individuals. Lower-income individuals defined in the present study are an economically disadvantaged group, as they possess income below the municipal tax exemption limit (the Ministry of Health, Labour and Welfare considers people with income below the municipal tax exemption limit to be low income people [29, 30]).

As health outcomes, we examined self-reported health. The literature indicates that self-reported health is a good predictor of objective health, including mortality and prevalence of various diseases [31]. The data on self-reported health were obtained from the Japan Gerontological Evaluation Study (JAGES) project,^5^ which conducts large-scale surveys among older people to investigate their living conditions (e.g., health status and participation in social groups) in more than 40 municipalities across Japan,^6^,^7^. In this municipality, the JAGES group conducted surveys in 2011 and 2013 among a random sample of functionally independent, community-dwelling individuals. The JAGES group distributed questionnaires to research subjects by mail. The response rates in 2011 and 2013 were 65.9% and 75.3%, respectively. In these surveys, self-reported health is measured by asking “What is your current health status: excellent, good, fair, or poor?” Based on this, we defined a dichotomous variable (1 = excellent/good, 0 = fair/poor)^8^.

Table 2 shows the summary statistics. To illustrate the healthcare utilization around the age threshold of 70 years, we reported the average health expenditure at ages 69 and 70, as well as the average health expenditure between the ages of 68 and 71. We had 1,420,252 person-month observations (representing 71,385 individuals) for health expenditure. Table 2 shows that health expenditure was lower before than after age 70. For example, the average outpatient expenditure per person-month for the overall sample increased from approximately 19,720 JPY ($183) to approximately 21,550 JPY ($200) at age 70. For self-reported health, we had 3,404 observations (representing 2,756 individuals). Most individuals in our sample reported their current health status as excellent/good. For example, about 82% of the overall sample aged 69 reported their current health status as excellent/good. Table 2 also shows that lower-income individuals aged 69 used somewhat more healthcare services, and they were less likely to report their health status as excellent/good than higher-income individuals. After the cost-sharing reduction, lower-income individuals aged 70 still used more healthcare services, but their self-reported health was similar to that of higher-income individuals.

**Table 2:** Summary statistics

	No. of observations	Mean at age 68–71	Mean at age 69	Mean at age 70
Panel A: Overall sample				
Health expenditure, JPY				
Outpatient care	1,420,252	21,000	19,719	21,554
Inpatient care	1,420,252	12,180	11,646	12,573
Self-reported health (excellent/good), %	3,404	85.6	82.3	86.6
Panel B: Lower-income individuals				
Health expenditure, JPY				
Outpatient care	331,032	22,974	22,049	22,934
Inpatient care	331,032	16,043	14,903	16,407
Self-reported health (excellent/good), %	736	83.7	78.1	86.0
Panel C: Middle-income individuals				
Health expenditure, JPY				
Outpatient care	542,501	20,200	18,899	20,744
Inpatient care	542,501	9,483	8,824	9,942
Self-reported health (excellent/good), %	1,338	87.1	84.0	87.5
Panel D: Higher-income individuals				
Health expenditure, JPY				
Outpatient care	546,719	20,600	19,155	21,506
Inpatient care	546,719	12,518	12,535	12,812
Self-reported health (excellent/good), %	1,330	85.2	82.7	85.9

Note: Health expenditure is in Japanese yen (JPY). 108 JPY was almost equal to $1 as of April 25, 2021

## Methods

We relied on a sharp RD design to identify the effect of cost-sharing on health expenditure and health, taking advantage of the reduction in the coinsurance rate from 30 to 10% at age 70. The unit of analysis was a person-month. We separately estimated the effects of reduced cost-sharing on outpatient care and inpatient care because the utilization decision for outpatient care and inpatient care can vary greatly; patients can freely decide whether to use outpatient care since there is no gatekeeping in Japan, while patients cannot be admitted to hospital without a physician’s agreement.

To examine the overall effect of the cost-sharing reduction on health expenditure, we estimated the following model for the overall sample using OLS:

1 $$Y_{it} = \beta_{0} + \beta_{1} Age70_{it} + f\left( a \right) + \beta_{2} Gender_{it} + \beta_{3} Income_{i} + Time_{t} + \varepsilon_{it} .{ }$$

where ${Y}_{it}$ is health expenditure of individual *i* at age in month *t*. ${Age70}_{it}$ is the dummy variable of interest, which equals one in the next month after individual *i* turns 70 and zero otherwise.$f(a)$ is the age trend in monthly age, which fully interacts with the Age70 dummy variable, allowing for different age trends before and after age 70. In our main analysis, we used a linear age trend, following the specification of recent studies [13, 18, 20]. In the sensitivity analysis, we also investigated the effect of the cost-sharing reduction using a quadratic age trend instead of a linear age trend. We adjusted for gender (${Gender}_{it}$), an indicator of the insurance premium category (${Income}_{i}$), and year–month fixed effects (${Time}_{t}$). $\beta$ is the parameter value to be estimated. Although our data had a panel structure, we did not include individual fixed effects in the model, because doing so is unnecessary for identification purposes [32].

Based on the idea of previous studies [e.g., 10, 20], we estimated a “donut hole” model, which excluded two months of data, namely, the months before and after an individual turns 70 years, from our regression analyses for health expenditure, as some individuals may postpone healthcare purchases until their coinsurance rate is reduced. Excluding these two-month data could mitigate upward bias (in absolute value) from such transitory responses. In the sensitivity analysis, we investigated whether our main results were sensitive to different sizes of donut holes.

When examining the heterogeneous effects of cost-sharing by income, we conducted stratified analyses by income. We divided the sample into three groups based on their income: lower-, middle-, and higher-income individuals, as shown in Table 1. We separately estimated the above model (Eq. (1)) for each income group.

For the health outcome, we did not use a “donut hole” model, but used full data because individuals are unlikely to intentionally delay the changes in their self-reported health due to the anticipation of the cost-sharing reduction. To examine the effects of the cost-sharing reduction on self-reported health, we estimated the following model using OLS:

2 $$H_{is} = \gamma_{0} + \gamma_{1} Age70_{is} + f\left( a \right) + \gamma_{2} Gender_{is} + \gamma_{3} Income_{i} + Survey year_{s} + \varepsilon_{is} .$$

where ${H}_{is}$ is self-reported health of individual *i* at age in survey year *s*. We used the same explanatory variables in Eq. (1) except that we adjusted for survey year fixed effects (${Survey year}_{s}$) instead of year-month fixed effects. We used a linear age trend in our main analysis and a quadratic age trend in the sensitivity analysis. $\gamma$ is the parameter value to be estimated. When examining the heterogeneous health effects by income, we conducted stratified analyses by income.

Finally, to assess the robustness of our main results, we performed a number of sensitivity analyses. First, we conducted a falsification test. We estimated Eq. (1) for those not subjected to the cost-sharing reduction (individuals in insurance premium categories 10–12). Second, to test whether our findings were sensitive to different specifications of age trends or different bandwidth choices, we used a quadratic age trend instead of a linear age trend or used a bandwidth of one year instead of two years. We also used a data-driven approach to choose the bandwidth [33]. We implemented a local linear regression with robust bias-corrected standard errors. Third, as we used income information in 2012, income categories may have been misclassified in 2013 and 2014. To test whether the potential misclassification affected our main results, we reanalyzed the data restricted to health expenditure in 2012. Fourth, to assess the robustness of our main results, we examined the effects of cost-sharing on health expenditures after additionally adjusting for individual fixed effects, although including individual fixed effects is unnecessary for identification purposes [32]. We also examined the effects of cost-sharing on health after additionally adjusting for individual characteristics, including education, family structure, and occupation (we did not include individual fixed effects for the analysis for health, because health outcomes were observed only once for some individuals). Fifth, because the distributions of outpatient and inpatient expenditure were highly skewed (Figures S1–S2 in the Supplementary Appendix), we investigated whether our main results for health expenditure were similar to estimates from a generalized linear model (GLM) with a log link and gamma distribution, which may better account for the highly skewed distributions. Sixth, to test whether our findings were sensitive to different sizes of donut holes around the age threshold of 70 years, we estimated the effect of cost-sharing, including two months of data before and after the age threshold and excluding four months of data before and after the age threshold, instead of excluding the two months of data. Finally, it is possible that individuals’ incomes changed at age 70 and the change in income may have biased our results. To test whether there is a discontinuity in income at age 70, we regressed indicators for lower-income or higher-income on the explanatory variables in Eq. (1) except the income category indicators. In this analysis, as we had income information only for 2012, we examined the association between income and age as of December 2012.

The analysis of the data and publication were approved by the Personal Information Protection Review Board of the municipality on October 6, 2015. Additionally, following the requirement from the municipality to protect the data, we conducted all data handling and analyses using a computer without an Internet connection in the city hall. This study was approved by the Ethics Committee of the Chiba University Graduate School of Medicine (approval No. 1777).

## Results

### Effects on outpatient care

**Fig. 1:**
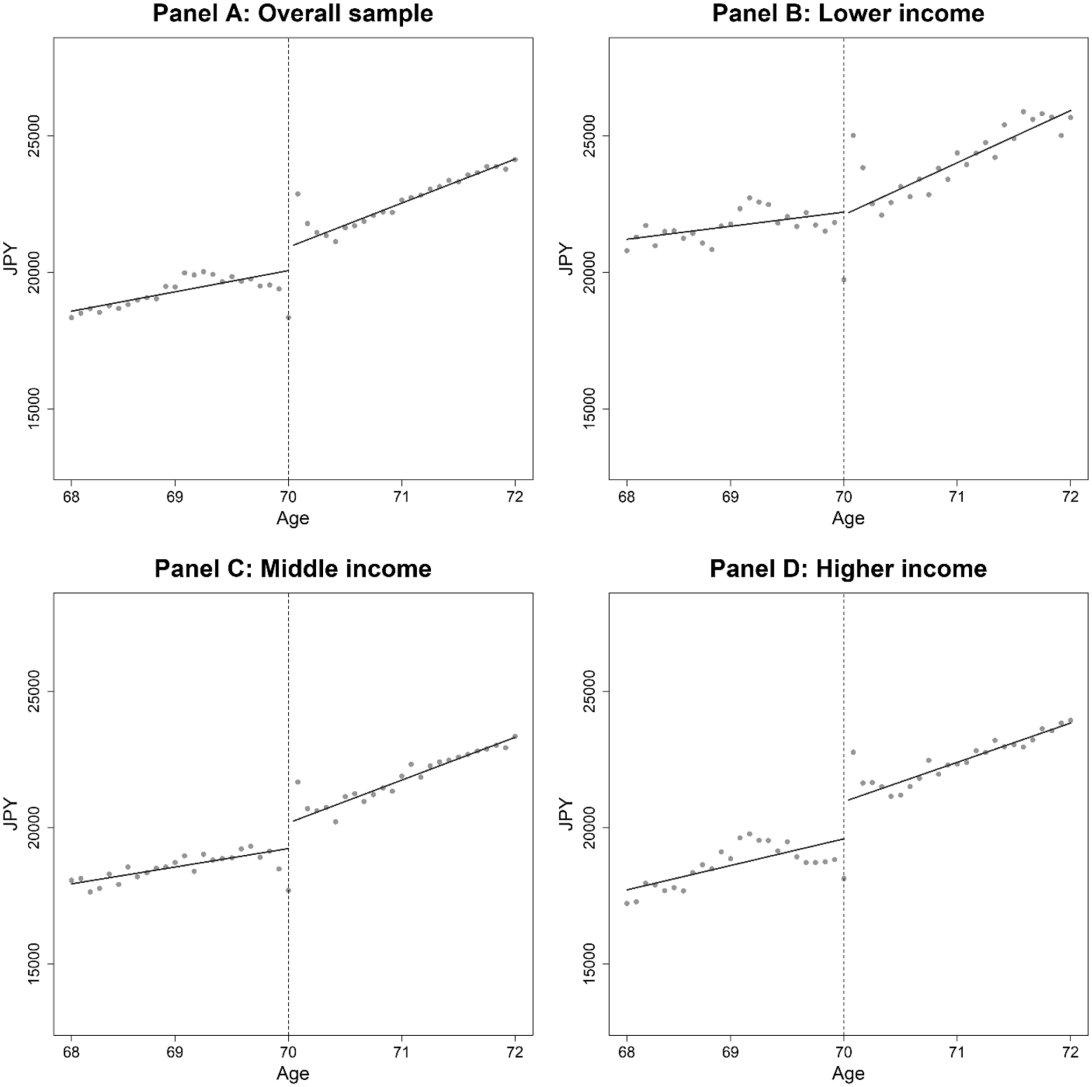
Outpatient expenditure by age (per person-month in JPY). Note: Dots represent the average health expenditure on outpatient care by age in months. The vertical dotted lines indicate the age threshold of 70 years. The coinsurance rate was 30% before age 70 and 10% after age 70. Dark lines are from fitting a linear function of age in month, separately for before and after age 70, excluding two months before and after the age threshold

We first report the graphical results before examining the effect of the cost-sharing reduction more formally in the regression analyses. In Fig. 1, we plotted the average health expenditure on outpatient care at each age (in months). Panel A of Fig. 1 shows that, for the overall sample, the outpatient expenditure per person steadily increased before age 70 and suddenly increased at age 70. Similar increases can be seen for middle- and higher-income individuals (Panels C and D), but there is no clear discontinuity at age 70 for lower-income individuals (Panel B). In addition, there is some evidence of transitory responses—outpatient expenditures dropped exactly one month before the age threshold of 70 years and greatly increased one month after the age threshold. This is consistent with our concern that individuals may postpone healthcare purchases until their coinsurance rate falls, supporting the exclusion of exactly two months before and after the age threshold from our regression analyses to avoid overestimating the effects of the cost-sharing reduction. Figure 1 also suggests that lower-income individuals may postpone the utilization to a large extent compared to other income groups.

In Table 3, the first panel presents the results from the RD regression for the overall sample. The “cost-sharing effect” column shows that reduced cost-sharing at age 70 increased the health expenditure on outpatient care by 947 JPY ($8.8) per person-month, which is equivalent to a 4.8% increase. The implied price elasticity for outpatient expenditure^9^ was -0.07.

**Table 3:** Effects of the cost-sharing reduction on the utilization of outpatient care

	Cost-sharing effect, JPY	Mean health expenditure at age 69, JPY	Difference in health expenditure, %	Elasticity
Panel A: Overall sample	947.4** (268.3)	19,719	4.8	− 0.07
Panel B: Lower income	− 21.4 (769.2)	22,049	− 0.1	0.001
Panel C: Middle income	1020.8** (370.8)	18,899	5.4	− 0.08
Panel D: Higher income	1466.3** (370.3)	19,155	7.7	− 0.11

Note: To save space, this table only reports the estimated coefficients for the RD dummy variables (1 = age 70 and above, 0 = otherwise). Full results are reported in Table S1 in the Supplementary Appendix. Robust standard errors corrected for clustering at the individual level are in parentheses. **: 1%, *: 5%

Next, we report the effect of the cost-sharing reduction on each income group. We found no evidence that reduced cost-sharing affected outpatient expenditure among lower-income individuals (Panel B of Table 3), consistent with Panel B of Fig. 1. In contrast, reduced cost-sharing significantly affected the utilization of outpatient care among middle- and higher-income individuals (Panels C and D of Table 3). The cost-sharing reduction increased outpatient expenditure by 1,021 JPY ($9.5), equivalent to a 5.4% increase, for middle-income individuals, and 1,470 JPY ($13.6), equivalent to a 7.7% increase, for higher-income individuals. The implied price elasticities for outpatient expenditure were almost zero, − 0.08, and − 0.11, for lower-, middle-, and higher-income individuals, respectively.

### Effects on inpatient care

**Fig. 2:**
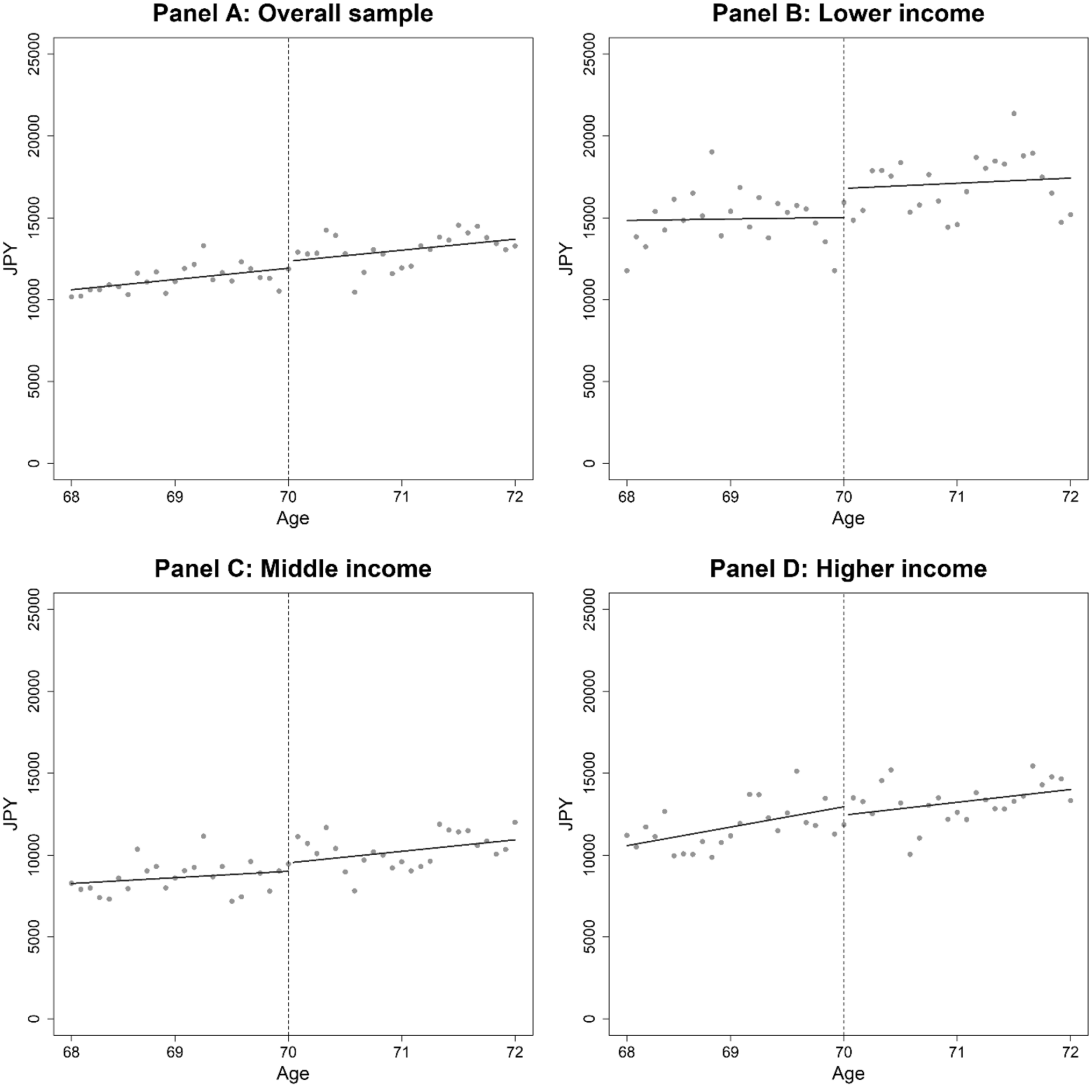
Inpatient expenditure by age (per person-month in JPY). Note: Dots represent the average health expenditure on inpatient care by age in months. The vertical dotted lines indicate the age threshold of 70 years. The coinsurance rate was 30% before age 70 and 10% after age 70. Dark lines are from fitting a linear function of age in month, separately for before and after age 70, excluding 2 months before and after the age threshold

Here, we provide results for inpatient care. In Fig. 2, we plotted the average health expenditure on inpatient care at each age (in months). In contrast to the drastic change at age 70 in the expenditure on outpatient care shown in Figs. 1, 2 reveals little visual evidence of discontinuities in inpatient expenditure for all income groups (Panels A–D).

Panel A of Table 4 indicates that there was no significant change in inpatient expenditure attributable to the cost-sharing reduction at age 70 for the overall sample. We also did not find any evidence that the cost-sharing reduction affected inpatient expenditure for lower-, middle-, and higher-income individuals (Panels B–D of Table 4).

**Table 4:** Effects of the cost-sharing reduction on the utilization of inpatient care

	Cost-sharing effect, JPY	Mean health expenditure at age 69, JPY	Difference in health expenditure, %	Elasticity
Panel A: Overall sample	505.0 (638.1)	11,646	4.3	− 0.07
Panel B: Lower income	1809.4 (1479.3)	14,903	12.1	− 0.18
Panel C: Middle income	648.0 (898.8)	8,824	7.3	− 0.11
Panel D: Higher income	-433.0 (1071.3)	12,535	− 3.5	0.05

Note: To save space, this table only reports the estimated coefficients for the RD dummy variables (1 = age 70 and above, 0 = otherwise). Full results are reported in Table S2 in the Supplementary Appendix. Robust standard errors corrected for clustering at the individual level are in parentheses. **: 1%, *: 5%

### Effects on health outcomes

**Fig. 3:**
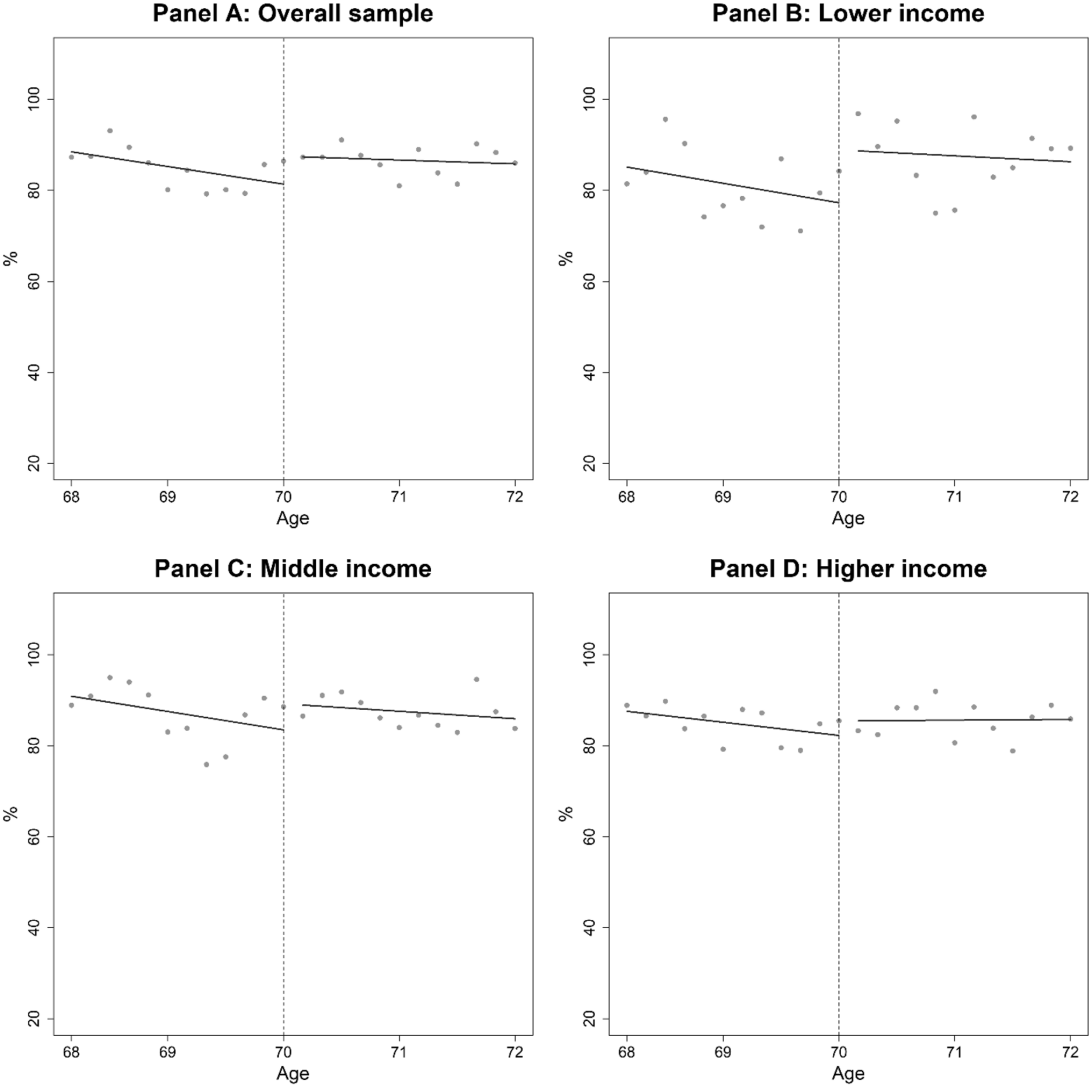
Proportion of excellent/good health status by age (per person per two months). Note: Dots represent the proportion of excellent or good health status by age. The vertical dotted lines indicate the age threshold of 70 years. The coinsurance rate was 30% before age 70 and 10% after age 70. Dark lines are from fitting a linear function of age in months, separately for before and after age 70

Figure 3 shows the relationship between age and self-reported health. We did not find any clear evidence that self-reported heath changed at age 70 when we focused on the overall sample (Panel A). We also did not observe any clear jumps at age 70 for middle- and higher-income individuals (Panels C and D). There may be some discontinuity at age 70 for lower-income individuals, but the effect is unclear due to the large variance (Panel B).

In Table 5, the first panel presents the results from the RD regression for the overall sample. The cost-sharing reduction significantly affected the probability of being in excellent/good health among the overall sample.

**Table 5:** Effects of the cost-sharing reduction on self-reported health

	Cost-sharing effect, %	Mean at age 69, %	Difference in health, %
Panel A: Overall sample	4.8* (2.3)	82.3	5.9
Panel B: Lower income	11.6* (5.0)	78.1	14.8
Panel C: Middle income	4.8 (3.5)	84.0	5.7
Panel D: Higher income	1.3 (3.9)	82.7	1.5

Note: To save space, this table only reports the estimated coefficients for the RD dummy variables (1 = age 70 and above, 0 = otherwise). Full results are reported in Table S3 in the Supplementary Appendix. Robust standard errors corrected for clustering at the individual level are in parentheses. **: 1%, *: 5%

Next, we present the health effect for each income group. For lower-income individuals, reduced cost-sharing significantly impacted self-reported health. Panel B of Table 5 indicates that the health status of approximately 12% of people improved following the cost-sharing reduction among lower-income individuals. However, the cost-sharing reduction did not significantly change self-reported health among middle- and higher-income individuals (Panels C and D of Table 5).

### Robustness check

When we estimated the same model for those not subjected to the cost-sharing reduction (individuals in insurance premium categories 10–12), we found no evidence that turning age 70 affected health expenditure and self-reported health among them (Tables S4–S5). These results substantially lessened the concern that another factor affects health expenditure and health status at age 70. Our main findings for outpatient care and self-reported health were unaffected by using a quadratic age trend instead of a linear age trend or by using a bandwidth of 1 year instead of 2 years (Tables S6–S7 and Tables S10–11). Our findings for inpatient care were somewhat sensitive to specifications of age trends and bandwidth choices (Tables S8–S9). We also used a data-driven approach to choose the bandwidth [33]. We used a local linear RD estimation with a triangular kernel. Our main findings for health expenditure were unaffected by using this approach (Tables S12–S13), and we found no evidence that reduced cost-sharing was associated with self-reported health (Table S14). Our main findings were not sensitive to including additional adjustment variables (Tables S15–S17). Additionally, our main findings for health expenditure were unaffected by using health expenditure data only for 2012 (Tables S18–S19); using a GLM model instead of OLS (Tables S20–S21); including 2 months of data before and after individuals turn 70 years (Tables S22–S23); and excluding 4 months of data before and after individuals turn 70 years (Tables S24–S25). Finally, we found no evidence that individuals changed their income at age 70 (Table S26 and Figure S3). These robustness checks provided additional confidence in our estimation results.

## Discussion

In the present study, we investigated how cost-sharing affects the utilization of healthcare and health among older people. We found that reduced cost-sharing modestly increased outpatient expenditure with a price elasticity of  – 0.07. Our estimate of the price elasticity for outpatient expenditure was slightly lower than the figures provided by previous studies of older people in Japan [9, 10] (approximately  – 0.20) and similar to figures from research for outpatient visits among older people in the U.S. [8] ( – 0.07 to  – 0.10). Although the existing studies focused only on a small set of services or on relatively wealthy older people [8–10], our estimate for outpatient care was within the range of similar estimates in the previous literature. We found no clear evidence that reduced cost-sharing affected inpatient expenditure, which is consistent with the findings of Fukushima et al. [10].

In addition to showing the overall effect of the cost-sharing reduction on older people, we examined the effects by income. To our knowledge, no previous studies have examined heterogeneous responses by income among older people in a creditable way. Our results show that the price elasticities for outpatient expenditure were almost zero,  – 0.08, and  – 0.11 for lower-, middle-, and higher-income individuals, respectively. This result suggests that lower-income individuals do not have a more elastic demand for outpatient care than higher-income individuals. Although our results may be a bit counterintuitive, several reasons may explain why high-income individuals had a more elastic demand for outpatient care. First, higher-income individuals, who tend to have higher education, may be able to take advantage of reduced cost-sharing due to better understanding of cost-sharing policy and better access of information and healthcare. A study in Japan found that highly educated men were more sensitive to the reduction of cost-sharing [34]. Second, higher-income individuals may be more likely to use price-sensitive healthcare services compared to lower-income individuals. Studies from developed countries found that higher income individuals used more elective healthcare services such as specialist visits [35, 36], which are sensitive to price changes [37]. Third, receiving healthcare may be less discretionary for lower-income individuals because they are generally sicker. Research found higher rates of chronic illness among low-income populations [38] and sicker patients have a less elastic demand for healthcare services [10, 17]. This may also explain why lower-income individuals seem to postpone the utilization to a larger extent compared to other income groups. For example, individuals with chronic illnesses, such as diabetes, may be able to delay regular visits for chronic disease management to some extent before the cost-sharing reduction. However, those with upper respiratory tract infections, for example, cannot postpone treatment too much, as they can often recover from these infections without seeing a physician. Because higher-income individuals are generally healthier, they may be more likely to use healthcare to treat these minor acute illnesses.

These reasons above are convincing, but cannot fully explain why our results differ from those of the two previous studies from Sweden [13, 18], which found that outpatient doctor visits by low-income children and young adults had a larger price response than those of their high-income counterparts. This clear contrast suggests that institutional reasons also matter. First, charged fees differ. In our settings, patients still have 10% coinsurance after the cost-sharing reduction, whereas the two previous studies examined the effect of free care. Evidence suggests that zero price is a special price, and people’s demand substantially increases when the price is zero [19, 39]. As lower-income individuals face a tighter budget constraint, even a 10% coinsurance rate may prevent lower-income individuals from increasing their utilization of outpatient care. Second, difference in gatekeeping exists. In the settings of the two previous studies in Sweden, there is a telephone triage system whereby patients must call a gatekeeping nurse and are only provided an appointment if deemed necessary by the gatekeeping nurse. Because there is no gatekeeping system in Japan, like in Korea and Taiwan, patients in Japan would find it easier to increase the utilization of low-value care, and high-income individuals may not hesitate to increase the utilization of low-value care, such as unnecessary specialist visits (studies found higher income individuals used more specialist visits [35, 36]), when cost-sharing is reduced.

Another important contribution of the present study is examining the effects of reduced cost-sharing on older people’s health. We found no evidence that reduced cost-sharing improved health outcomes among middle- and higher-income individuals, but found that it significantly improved self-reported health among lower-income individuals. While our regression analysis shows that there was a statistically significant difference, we believe that, when employing an RD design, we need clear graphical evidence of a discontinuity to convince readers. Therefore, we concluded that, although lower-income individuals might derive health benefits from the cost-sharing reduction, drawing clear conclusions about health outcomes is difficult because of the lack of strong graphical evidence to support health improvement.

Our study has several important implications for cost-sharing policies. First, we found that the response to reduced cost-sharing for outpatient care was driven by middle- and higher-income individuals. Our results offer a rationale for reducing cost-sharing among older people with lower income because the cost-sharing reduction would lessen the financial risk faced by them and would not increase additional health expenditure. Second, we found that health improvement caused by reduced cost-sharing, if any, may be driven by lower-income individuals, not middle- and higher-income individuals. This result suggests that the increase in health expenditure among middle- and higher-income individuals may not be associated with improved health. Taken together, varying cost-sharing by income (i.e., smaller cost-sharing for lower-income individuals and larger cost-sharing for higher-income individuals) for older people may sufficiently prevent the overuse of outpatient care without compromising health.

Our study has several limitations. First, our data were collected from one municipality in Japan. Therefore, our findings may not be generalized to other populations, although this municipality is a typical city in Japan in terms of the proportion of older people and the distribution of income-related insurance premium groups. Thus, further research is warranted to understand the overall and heterogeneous effects of cost-sharing among other age groups and regions. Second, our analysis excluded very low-income (i.e., the bottom 25% of the income distribution) and very high-income (i.e., the top 16% of the income distribution) individuals because their cost-sharing did not change at age 70. Therefore, our findings may not be generalizable to other income groups. However, note that we found a heterogenous impact of cost-sharing by income even after excluding very low- and very high-income individuals, suggesting our findings may be lower-bound estimates of the heterogeneous effects by income. Third, we could not draw clear conclusions about health outcomes because of limited sample size. Thus, future research should investigate health impacts of cost-sharing using larger datasets. Fourth, we could not identify the mechanisms behind the heterogeneous effects of cost-sharing. Thus, further research is required to investigate mechanisms through which lower-income individuals have a smaller price response than higher-income ones. Finally, we applied the income categories for 2012 to all years. Therefore, our income categories may have been misclassified in 2013 and 2014. However, our main findings were not changed when we used the data on health expenditure only for 2012.

## Conclusions

Using large administrative claims data, we estimated the effects of cost-sharing on the utilization of healthcare among older people. We found that reduced cost-sharing modestly increased outpatient expenditure with a price elasticity of  – 0.07. When examining the effects of reduced cost-sharing by income, we found that the price elasticities for outpatient expenditure were almost zero,  – 0.08, and  – 0.11 for lower-, middle-, and higher-income individuals, respectively. Our results offer a rationale for reducing cost-sharing among older people with lower income since the cost-sharing reduction would lessen their financial risk and would not increase additional health expenditure. Varying cost-sharing by income (i.e., smaller cost-sharing for lower-income individuals and larger cost-sharing for higher-income individuals) for older people may sufficiently prevent the overuse of outpatient care without compromising health.

## Supplementary Information

Below is the link to the electronic supplementary material.

Supplementary file1 (DOCX 388 KB)

## Data Availability

No additional data available.

## Footnotes

1 The average income included not only older people but also other adults.

2 Individuals on public assistance are covered by the public subsidy and are exempt from the premium contribution and cost-sharing.

3 Because our enrollment data were for long-term care insurance, not for community-based public health insurance, these data were incomplete. All older people in the municipality enroll in long-term care insurance, but older people who enroll in employee-based health insurance do not enroll in community-based public health insurance. This may lead to bias our results, especially when older people retire at age 70 (i.e., if older people retire at age 70 and they switch from employee-based health insurance to community-based public health insurance at age 70, this would increase health expenditure of community-based public health insurance). However, as explained in Sect. 3, our case has little concern because the mandatory retirement age in most firms is not 70 years.

4 We excluded 1% of the person-month observations from our sample because their income information was missing.

5 For more details on the JAGES project, see https://www.jages.net/.

6 Merging the enrollment information with the JAGES survey, we calculated the ages of the JAGES survey respondents.

7 Merging the insurance premium category data with the JAGES survey, we divided respondents into the three income groups in the same way as explained above.

8 We excluded 0.9% of the observations from our sample because their income information was missing. We also excluded 2.5% of the observations from our sample because their self-reported health data were missing.

9 In the present study, we obtained the elasticity as follows: elasticity = ($\Delta$ Q/Q)/ ($\Delta$ P/P) = (the cost-sharing effect/the mean health expenditure at age 69)/(the average cost-sharing after age 70/the average cost-sharing before age 70 − 1).

## Code availability

No code available.
