# Supplementary material for: The effects of patient cost-sharing on health expenditure and health among older people: Heterogeneity across income groups
Source: Eur J Health Econ. 2021 Nov 15;23(5):847–61. doi: 10.1007/s10198-021-01399-6 (PMC9170661; doi:10.1007/s10198-021-01399-6)
Supplement: Supplementary file 1 — Supplementary file1 (DOCX 388 KB) [file 10198_2021_1399_MOESM1_ESM.docx]

**Supplementary Appendix**

**The effects of patient cost-sharing on health expenditure and health among older people: Heterogeneity across income groups**

[**A. Stop-loss** 3](#_Toc80860480)

[**Table S1.** Effects of the cost-sharing reduction on the utilization of outpatient care (full results of Table 3). 5](#_Toc80860481)

[**Table S2.** Effects of the cost-sharing reduction on the utilization of inpatient care (full results of Table 4). 6](#_Toc80860482)

[**Table S3.** Effects of the cost-sharing reduction on self-reported health (full results of Table 5). 7](#_Toc80860483)

[**Table S4**. Effects of turning 70 years on health expenditure, among people not subjected to the cost-sharing reduction. 8](#_Toc80860484)

[**Table S5.** Effect of turning 70 years on self-reported health, among people not subjected to the cost-sharing reduction. 9](#_Toc80860485)

[**Table S6.** Effects of the cost-sharing reduction on the utilization of outpatient care, using a quadratic age trend. 10](#_Toc80860486)

[**Table S7.** Effects of the cost-sharing reduction on the utilization of outpatient care, using one-year window. 11](#_Toc80860487)

[**Table S8**. Effects of the cost-sharing reduction on the utilization of inpatient care, using a quadratic age trend. 12](#_Toc80860488)

[**Table S9**. Effects of the cost-sharing reduction on the utilization of inpatient care, using one-year window. 13](#_Toc80860489)

[**Table S10.** Effects of the cost-sharing reduction on self-reported health, using a quadratic age trend. 14](#_Toc80860490)

[**Table S11.** Effects of the cost-sharing reduction on self-reported health, using one-year window. 15](#_Toc80860491)

[**Table S12.** Effects of the cost-sharing reduction on the utilization of outpatient care, using local linear regression. 16](#_Toc80860492)

[**Table S13.** Effects of the cost-sharing reduction on the utilization of inpatient care, using local linear regression. 17](#_Toc80860493)

[**Table S14**. Effects of the cost-sharing reduction on self-reported health, using local linear regression. 18](#_Toc80860494)

[**Table S15.** Effects of the cost-sharing reduction on the utilization of outpatient care, additionally adjusting for individual fixed effects. 19](#_Toc80860495)

[**Table S16.** Effects of the cost-sharing reduction on the utilization of inpatient care, additionally adjusting for individual fixed effects. 20](#_Toc80860496)

[**Table S17.** Effects of the cost-sharing reduction on self-reported health, additionally adjusting for individual characteristics. 21](#_Toc80860497)

[**Table S18.** Effects of the cost-sharing reduction on the utilization of outpatient care, using data for 2012. 22](#_Toc80860498)

[**Table S19.** Effects of the cost-sharing reduction on the utilization of inpatient care, using data for 2012. 23](#_Toc80860499)

[**Table S20.** Effects of the cost-sharing reduction on the utilization of outpatient care, using a generalized linear model. 24](#_Toc80860500)

[**Table S21.** Effects of the cost-sharing reduction on the utilization of inpatient care, using a generalized linear model. 25](#_Toc80860501)

[**Table S22.** Effects of the cost-sharing reduction on the utilization of outpatient care, including two months of data before and after individuals turn 70 years. 26](#_Toc80860502)

[**Table S23.** Effects of the cost-sharing reduction on the utilization of inpatient care, including two months of data before and after individuals turn 70 years. 27](#_Toc80860503)

[**Table S24.** Effects of the cost-sharing reduction on the utilization of outpatient care, excluding four months of data before and after individuals turn 70 years. 28](#_Toc80860504)

[**Table S25.** Effects of the cost-sharing reduction on the utilization of inpatient care, excluding four months of data before and after individuals turn 70 years. 29](#_Toc80860505)

[**Table S26.** Effect of turning 70 years on income. 30](#_Toc80860506)

[**Figure S1.** Distribution of outpatient expenditure. 31](#_Toc80860507)

[**Figure S2.** Distribution of inpatient expenditure. 32](#_Toc80860508)

[**Figure S3.** Proportions of lower-income or higher-income individuals by age. 33](#_Toc80860509)

[**References** 34](#_Toc80860510)

# **A. Stop-loss**

There is a stop-loss in Japanese public health insurance system, to protect patients against catastrophic health expenditure; when the monthly out-of-pocket payment exceeds a threshold value, cost-sharing is reduced, as described in Table 1. This stop-loss may pose a challenge for calculating demand elasticity if patients are forward-looking and know with certainty that their out-of-pocket payment is likely to exceed the threshold. However, the effect of the stop-loss on overutilization would be much smaller in our case, for the following two reasons as well-discussed in Fukushima et al. (2016) and Shigeoka (2014) [1, 2]. First, very few patients exceeded the threshold. For example, only 1.3% of enrollee-months exceeded the threshold value in our data for those aged 69 years. Second, the stop-loss is set monthly in Japan, unlike in the U.S., where the stop-loss is set annually. This shorter interval may make it more difficult for patients to overuse healthcare. In addition, evidence suggests that even under an annual stop-loss, people myopically responded to the stop-loss [3].

Nonetheless, to take the stop-loss into account, we calculated the average cost-sharing percentages before and after 70 years. First, we used our data of individuals aged 69 and calculated the actual cost-sharing percentage for each individual at age 69, based on the rules in Table 1. Next, we computed the hypothetical cost-sharing percentages for those above age 70, using the same data on those aged 69 years, under the assumption that they were subjected to the cost-sharing rule above age 70. We then took the average cost-sharing percentages. From the above calculation, the average cost-sharing percentages before and after 70 years were 29.3% and 9.8% for the overall sample; 28.6% and 9.6% for lower-income individuals; 29.6% and 9.9% for middle-income individuals; and 29.5% and 9.8% for higher-income individuals, respectively. These figures were close to 30% and 10%, respectively (the coinsurance rates without the stop-loss). This is not surprising because few patients qualified for the stop-loss. We used these calculated figures when estimating the price elasticity.

# **Table S1.** Effects of the cost-sharing reduction on the utilization of outpatient care (full results of Table 3).

|  | 1 | 2 | 3 | 4 |
| --- | --- | --- | --- | --- |
|  | Overall sample | Lower income | Middle income | Higher income |
| Age70 | 947.4** | -21.4 | 1020.8** | 1466.3** |
|  | (268.3) | (769.2) | (370.8) | (370.3) |
| Age | 55.4** | 34.9 | 52.7** | 69.7** |
|  | (14.8) | (44.0) | (20.4) | (19.6) |
| Age*Age70 | 73.1** | 118.7* | 76.0* | 43.8 |
|  | (22.1) | (57.8) | (33.5) | (31.3) |
| Gender | -3148.9** | -4079.3** | -2457.2* | -2801.9** |
| (Reference: Male) | (410.2) | (889.9) | (1036.2) | (414.7) |
| Income |  |  |  |  |
| Category 3 | Reference | Reference |  |  |
|  |  |  |  |  |
| Category 4 | 2033.6** | 1645.3* |  |  |
|  | (714.8) | (824.0) |  |  |
| Category 5 | -735.9 |  | Reference |  |
|  | (511.3) |  |  |  |
| Category 6 | -319.0 |  | 494.7 |  |
|  | (614.0) |  | (504.7) |  |
| Category 7 | -2464.9** |  |  | Reference |
|  | (614.0) |  |  |  |
| Category 8 | -2040.0** |  |  | 481.3 |
|  | (702.8) |  |  | (422.4) |
| Include year-month fixed effects | Yes | Yes | Yes | Yes |
| Constant | 20405.1** | 20959.7** | 19378.7** | 17576.6** |
|  | (748.4) | (1401.8) | (1098.3) | (442.3) |

Note: Column 1 presents the full results from the RD regression for the overall sample. Columns 2-4 present the results for lower-, middle-, and higher-income individuals, respectively. Robust standard errors corrected for clustering at the individual level are in parentheses. **: 1%, *: 5%.

# **Table S2.** Effects of the cost-sharing reduction on the utilization of inpatient care (full results of Table 4).

|  | 1 | 2 | 3 | 4 |
| --- | --- | --- | --- | --- |
|  | Overall sample | Lower income | Middle income | Higher income |
| Age70 | 505.0 | 1809.4 | 648.0 | -433.0 |
|  | (638.1) | (1479.3) | (898.8) | (1071.3) |
| Age | 37.1 | -13.7 | 23.4 | 83.4 |
|  | (31.6) | (73.3) | (44.6) | (53.5) |
| Age*Age70 | 5.0 | 30.3 | 29.2 | -34.2 |
|  | (48.0) | (112.6) | (68.4) | (79.2) |
| Gender | -10160.0** | -12652.8** | -10696.6** | -8200.0** |
| (Reference: Male) | (554.3) | (1135.7) | (1444.2) | (610.8) |
| Income |  |  |  |  |
| Category 3 | Reference | Reference |  |  |
|  |  |  |  |  |
| Category 4 | 1242.1 | 156.0 |  |  |
|  | (1005.4) | (1055.6) |  |  |
| Category 5 | -2288.5** |  | Reference |  |
|  | (705.4) |  |  |  |
| Category 6 | -2489.2** |  | -257.9 |  |
|  | (745.5) |  | (551.1) |  |
| Category 7 | -5798.3** |  |  | Reference |
|  | (782.1) |  |  |  |
| Category 8 | -6976.5** |  |  | -868.2 |
|  | (914.8) |  |  | (679.2) |
| Include year-month fixed effects | Yes | Yes | Yes | Yes |
| Constant | 17905.9** | 18047.0** | 16824.0** | 12001.0** |
|  | (1013.1) | (1926.1) | (1717.0) | (1146.0) |

Note: Column 1 presents the full results from the RD regression for the overall sample. Columns 2-4 present the results for lower-, middle-, and higher-income individuals, respectively. Robust standard errors corrected for clustering at the individual level are in parentheses. **: 1%, *: 5%.

# **Table S3.** Effects of the cost-sharing reduction on self-reported health (full results of Table 5).

|  | 1 | 2 | 3 | 4 |
| --- | --- | --- | --- | --- |
|  | Overall sample | Lower income | Middle income | Higher income |
| Age70 | 4.8* | 11.6* | 4.8 | 1.3 |
|  | (2.3) | (5.0) | (3.5) | (3.9) |
| Age | -0.2* | -0.4 | -0.2 | -0.2 |
|  | (0.1) | (0.3) | (0.2) | (0.2) |
| Age*Age70 | 0.2 | 0.2 | 0.1 | 0.3 |
|  | (0.2) | (0.3) | (0.3) | (0.3) |
| Gender | 8.4** | 11.9** | 8.7* | 6.4** |
| (Reference: Male) | (1.8) | (3.8) | (3.9) | (2.2) |
| Income |  |  |  |  |
| Category 3 | Reference | Reference |  |  |
|  |  |  |  |  |
| Category 4 | -0.9 | 0.5 |  |  |
|  | (3.1) | (3.6) |  |  |
| Category 5 | -1.0 |  | Reference |  |
|  | (2.4) |  |  |  |
| Category 6 | 1.7 |  | 2.7 |  |
|  | (2.4) |  | (2.0) |  |
| Category 7 | 1.9 |  |  | Reference |
|  | (2.6) |  |  |  |
| Category 8 | 7.1* |  |  | 5.0* |
|  | (2.8) |  |  | (2.1) |
| Survey year | -1.6 | 0.3 | -2.0 | -2.4 |
| (Reference: 2011) | (1.1) | (2.4) | (1.7) | (1.8) |
| Constant | 76.3** | 69.1** | 75.9** | 80.7 |
|  | (3.0) | (5.1) | (4.8) | (3.1) |

Note: Column 1 presents the full results from the RD regression for the overall sample. Columns 2-4 present the results for lower-, middle-, and higher-income individuals, respectively. Robust standard errors corrected for clustering at the individual level are in parentheses. **: 1%, *: 5%.

# **Table S4**. Effects of turning 70 years on health expenditure, among people not subjected to the cost-sharing reduction.

|  | Coef. |
| --- | --- |
| **Outpatient expenditure, JPY** | 41.7 |
|  | (755.9) |
|  |  |
| **Inpatient expenditure, JPY** | -2518.8 |
|  | (2156.9) |

Note: To save space, this table only reports the estimated coefficients for the RD dummy variables (1=age 70 and above, 0=otherwise). Robust standard errors corrected for clustering at the individual level are in parentheses. **: 1%, *: 5%.

# **Table S5.** Effect of turning 70 years on self-reported health, among people not subjected to the cost-sharing reduction.

|  | Coef. |
| --- | --- |
| **Self-reported health, %** | 3.7 |
|  | (5.8) |

Note: To save space, this table only reports the estimated coefficients for the RD dummy variables (1=age 70 and above, 0=otherwise). Robust standard errors corrected for clustering at the individual level are in parentheses. **: 1%, *: 5%.

# **Table S6.** Effects of the cost-sharing reduction on the utilization of outpatient care, using a quadratic age trend.

|  | Cost-sharing effect, JPY | Mean health expenditure at age 69, JPY | Difference in health expenditure, % | Elasticity |
| --- | --- | --- | --- | --- |
| **Panel A: Overall sample** | 1612.3** | 19,719 | 8.2 | -0.12 |
|  | (274.5) |  |  |  |
|  |  |  |  |  |
| **Panel B: Lower income** | 536.7 | 22,049 | 2.4 | -0.04 |
|  | (632.2) |  |  |  |
|  |  |  |  |  |
| **Panel C: Middle income** | 1201.1** | 18,899 | 6.4 | -0.10 |
|  | (425.7) |  |  |  |
|  |  |  |  |  |
| **Panel D: Higher income** | 2665.9** | 19,155 | 13.9 | -0.21 |
|  | (427.9) |  |  |  |

Note: To save space, this table only reports the estimated coefficients for the RD dummy variables (1=age 70 and above, 0=otherwise). Robust standard errors corrected for clustering at the individual level are in parentheses. **: 1%, *: 5%.

# **Table S7.** Effects of the cost-sharing reduction on the utilization of outpatient care, using one-year window.

|  | Cost-sharing effect, JPY | Mean health expenditure at age 69, JPY | Difference in health expenditure, % | Elasticity |
| --- | --- | --- | --- | --- |
| **Panel A: Overall sample** | 1708.2** | 19,719 | 8.7 | -0.13 |
|  | (240.0) |  |  |  |
|  |  |  |  |  |
| **Panel B: Lower income** | 872.9 | 22,049 | 4.0 | -0.06 |
|  | (539.3) |  |  |  |
|  |  |  |  |  |
| **Panel C: Middle income** | 1285.0** | 18,899 | 6.8 | -0.10 |
|  | (374.8) |  |  |  |
|  |  |  |  |  |
| **Panel D: Higher income** | 2634.6** | 19,155 | 13.8 | -0.21 |
|  | (379.4) |  |  |  |

Note: To save space, this table only reports the estimated coefficients for the RD dummy variables (1=age 70 and above, 0=otherwise). Robust standard errors corrected for clustering at the individual level are in parentheses. **: 1%, *: 5%.

# **Table S8**. Effects of the cost-sharing reduction on the utilization of inpatient care, using a quadratic age trend.

|  | Cost-sharing effect, JPY | Mean health expenditure at age 69, JPY | Difference in health expenditure, % | Elasticity |
| --- | --- | --- | --- | --- |
| **Panel A: Overall sample** | 2047.2* | 11,646 | 17.6 | -0.26 |
|  | (945.5) |  |  |  |
|  |  |  |  |  |
| **Panel B: Lower income** | 3727.3 | 14,903 | 25.0 | -0.38 |
|  | (2117.2) |  |  |  |
|  |  |  |  |  |
| **Panel C: Middle income** | 2562.7 | 8,824 | 29.0 | -0.44 |
|  | (1334.8) |  |  |  |
|  |  |  |  |  |
| **Panel D: Higher income** | 577.5 | 12,535 | 4.6 | -0.07 |
|  | (1622.9) |  |  |  |

Note: To save space, this table only reports the estimated coefficients for the RD dummy variables (1=age 70 and above, 0=otherwise). Robust standard errors corrected for clustering at the individual level are in parentheses. **: 1%, *: 5%.

# **Table S9**. Effects of the cost-sharing reduction on the utilization of inpatient care, using one-year window.

|  | Cost-sharing effect, JPY | Mean health expenditure at age 69, JPY | Difference in health expenditure, % | Elasticity |
| --- | --- | --- | --- | --- |
| **Panel A: Overall sample** | 2592.1** | 11,646 | 22.3 | -0.33 |
|  | (864.5) |  |  |  |
|  |  |  |  |  |
| **Panel B: Lower income** | 4535.5* | 14,903 | 30.4 | -0.46 |
|  | (1909.1) |  |  |  |
|  |  |  |  |  |
| **Panel C: Middle income** | 2635.8* | 8,824 | 29.9 | -0.45 |
|  | (1206.7) |  |  |  |
|  |  |  |  |  |
| **Panel D: Higher income** | 1400.0 | 12,535 | 11.2 | -0.17 |
|  | (1507.9) |  |  |  |

Note: To save space, this table only reports the estimated coefficients for the RD dummy variables (1=age 70 and above, 0=otherwise). Robust standard errors corrected for clustering at the individual level are in parentheses. **: 1%, *: 5%.

# **Table S10.** Effects of the cost-sharing reduction on self-reported health, using a quadratic age trend.

|  | Cost-sharing effect, % | Mean at age 69, % | Difference in health, % |
| --- | --- | --- | --- |
| **Panel A: Overall sample** | 4.4 | 82.3 | 5.3 |
|  | (3.4) |  |  |
|  |  |  |  |
| **Panel B: Lower income** | 18.1** | 78.1 | 23.2 |
|  | (6.7) |  |  |
|  |  |  |  |
| **Panel C: Middle income** | 2.3 | 84.0 | 2.7 |
|  | (5.3) |  |  |
|  |  |  |  |
| **Panel D: Higher income** | -1.3 | 82.7 | -1.6 |
|  | (5.6) |  |  |

Note: To save space, this table only reports the estimated coefficients for the RD dummy variables (1=age 70 and above, 0=otherwise). Robust standard errors corrected for clustering at the individual level are in parentheses. **: 1%, *: 5%.

# **Table S11.** Effects of the cost-sharing reduction on self-reported health, using one-year window.

|  | Cost-sharing effect, % | Mean at age 69, % | Difference in health, % |
| --- | --- | --- | --- |
| **Panel A: Overall sample** | 4.5 | 82.3 | 4.0 |
|  | (3.2) |  |  |
|  |  |  |  |
| **Panel B: Lower income** | 20.2** | 78.1 | 19.2 |
|  | (6.3) |  |  |
|  |  |  |  |
| **Panel C: Middle income** | 0.8 | 84.0 | 1.8 |
|  | (4.8) |  |  |
|  |  |  |  |
| **Panel D: Higher income** | -0.5 | 82.7 | -1.8 |
|  | (5.4) |  |  |

Note: To save space, this table only reports the estimated coefficients for the RD dummy variables (1=age 70 and above, 0=otherwise). Robust standard errors corrected for clustering at the individual level are in parentheses. **: 1%, *: 5%.

# **Table S12.** Effects of the cost-sharing reduction on the utilization of outpatient care, using local linear regression.

|  | Cost-sharing effect, JPY | Mean health expenditure at age 69, JPY | Difference in health expenditure, % | Elasticity |
| --- | --- | --- | --- | --- |
| **Panel A: Overall sample** | 2765.7** | 19,719 | 14.0 | -0.21 |
|  | (482.5) |  |  |  |
|  |  |  |  |  |
| **Panel B: Lower income** | 2155.9 | 22,049 | 9.8 | -0.15 |
|  | (1249.7) |  |  |  |
|  |  |  |  |  |
| **Panel C: Middle income** | 2259.5** | 18,899 | 12.0 | -0.18 |
|  | (626.6) |  |  |  |
|  |  |  |  |  |
| **Panel D: Higher income** | 3281.3** | 19,155 | 17.1 | -0.26 |
|  | (662.8) |  |  |  |

Note: To save space, this table only reports the estimated coefficients for the RD dummy variables (1=age 70 and above, 0=otherwise). We used a local linear RD estimation with a triangular kernel. We used a data-driven approach to choose a bandwidth, which yielded an optimal bandwidth of 7.8, 10.0, 8.8, and 9.7 months for overall sample, lower-income individuals, middle-income individuals, and higher-income individuals, respectively. Robust standard errors corrected for clustering at the individual level are in parentheses. **: 1%, *: 5%.

# **Table S13.** Effects of the cost-sharing reduction on the utilization of inpatient care, using local linear regression.

|  | Cost-sharing effect, JPY | Mean health expenditure at age 69, JPY | Difference in health expenditure, % | Elasticity |
| --- | --- | --- | --- | --- |
| **Panel A: Overall sample** | 3357.1* | 11,646 | 28.8 | -0.43 |
|  | (1485.8) |  |  |  |
|  |  |  |  |  |
| **Panel B: Lower income** | 5474.5 | 14,903 | 36.7 | -0.55 |
|  | (3216.5) |  |  |  |
|  |  |  |  |  |
| **Panel C: Middle income** | 2629.5 | 8,824 | 29.8 | -0.45 |
|  | (2035.1) |  |  |  |
|  |  |  |  |  |
| **Panel D: Higher income** | 2984.5 | 12,535 | 23.8 | -0.36 |
|  | (2543.0) |  |  |  |

Note: To save space, this table only reports the estimated coefficients for the RD dummy variables (1=age 70 and above, 0=otherwise). We used a local linear RD estimation with a triangular kernel. We used a data-driven approach to choose a bandwidth, which yielded an optimal bandwidth of 8.6, 9.0, 9.3, and 8.5 months for overall sample, lower-income individuals, middle-income individuals, and higher-income individuals, respectively. Robust standard errors corrected for clustering at the individual level are in parentheses. **: 1%, *: 5%.

# **Table S14**. Effects of the cost-sharing reduction on self-reported health, using local linear regression.

|  | Cost-sharing effect, % | Mean at age 69, % | Difference in health, % |
| --- | --- | --- | --- |
| **Panel A: Overall sample** | -1.7 | 82.3 | -2.1 |
|  | (4.0) |  |  |
|  |  |  |  |
| **Panel B: Lower income** | 14.3 | 78.1 | 18.3 |
|  | (8.2) |  |  |
|  |  |  |  |
| **Panel C: Middle income** | -2.6 | 84.0 | -3.1 |
|  | (8.0) |  |  |
|  |  |  |  |
| **Panel D: Higher income** | -6.2 | 82.7 | -7.5 |
|  | (7.8) |  |  |

Note: To save space, this table only reports the estimated coefficients for the RD dummy variables (1=age 70 and above, 0=otherwise). We used a local linear RD estimation with a triangular kernel. We used a data-driven approach to choose a bandwidth, which yielded an optimal bandwidth of 8.6, 9.9, 8.9, and 9.0 months for overall sample, lower-income individuals, middle-income individuals, and higher-income individuals, respectively. Robust standard errors corrected for clustering at the individual level are in parentheses. **: 1%, *: 5%.

# **Table S15.** Effects of the cost-sharing reduction on the utilization of outpatient care, additionally adjusting for individual fixed effects.

|  | Cost-sharing effect, JPY | Mean health expenditure at age 69, JPY | Difference in health expenditure, % | Elasticity |
| --- | --- | --- | --- | --- |
| Panel A: Overall sample | 1256.2** | 19,719 | 6.4 | -0.10 |
|  | (223.1) |  |  |  |
|  |  |  |  |  |
| Panel B: Lower income | 829.2 | 22,049 | 3.8 | -0.06 |
|  | (587.0) |  |  |  |
|  |  |  |  |  |
| Panel C: Middle income | 1162.6** | 18,899 | 6.2 | -0.09 |
|  | (315.7) |  |  |  |
|  |  |  |  |  |
| Panel D: Higher income | 1608.7** | 19,155 | 8.4 | -0.13 |
|  | (337.3) |  |  |  |

Note: To save space, this table only reports the estimated coefficients for the RD dummy variables (1=age 70 and above, 0=otherwise). Robust standard errors corrected for clustering at the individual level are in parentheses. **: 1%, *: 5%.

# **Table S16.** Effects of the cost-sharing reduction on the utilization of inpatient care, additionally adjusting for individual fixed effects.

|  | Cost-sharing effect, JPY | Mean health expenditure at age 69, JPY | Difference in health expenditure, % | Elasticity |
| --- | --- | --- | --- | --- |
| Panel A: Overall sample | 158.7 | 11,646 | 1.4 | -0.02 |
|  | (663.5) |  |  |  |
|  |  |  |  |  |
| Panel B: Lower income | 1681.6 | 14,903 | 11.3 | -0.17 |
|  | (1508.4) |  |  |  |
|  |  |  |  |  |
| Panel C: Middle income | -45.0 | 8,824 | -0.5 | 0.01 |
|  | (954.3) |  |  |  |
|  |  |  |  |  |
| Panel D: Higher income | -572.6 | 12,535 | -4.6 | 0.07 |
|  | (1112.9) |  |  |  |

Note: To save space, this table only reports the estimated coefficients for the RD dummy variables (1=age 70 and above, 0=otherwise). Robust standard errors corrected for clustering at the individual level are in parentheses. **: 1%, *: 5%.

# **Table S17.** Effects of the cost-sharing reduction on self-reported health, additionally adjusting for individual characteristics.

|  | Cost-sharing effect, % | Mean at age 69, % | Difference in health, % |
| --- | --- | --- | --- |
| Panel A: Overall sample | 6.0* | 82.3 | 7.3 |
|  | (2.5) |  |  |
|  |  |  |  |
| Panel B: Lower income | 13.9* | 78.1 | 17.8 |
|  | (5.5) |  |  |
|  |  |  |  |
| Panel C: Middle income | 7.2 | 84.0 | 8.6 |
|  | (3.8) |  |  |
|  |  |  |  |
| Panel D: Higher income | 2.8 | 82.7 | 3.4 |
|  | (4.1) |  |  |

Note: To save space, this table only reports the estimated coefficients for the RD dummy variables (1=age 70 and above, 0=otherwise). Robust standard errors corrected for clustering at the individual level are in parentheses. For this analysis, we additionally adjusted for individual characteristics, including education (<6 years, 6-9 years, 10-12 years, ≥13 years, others), family structure (marital status [married, widowed, divorced, unmarried, others] and fixed effects for the number of household members), and occupation (working status [currently working, retired, no work experience] and size of the company individuals worked for the longest [<10 workers, 10-49 workers, 50-499 workers, 500-9999 workers, ≥10000 workers, not sure, no work experience]). However, due to missing data on the individual characteristics, we excluded 529, 124, 267, and 138 observations for the overall, lower-, middle-, and higher-income individuals, respectively. **: 1%, *: 5%.

# **Table S18.** Effects of the cost-sharing reduction on the utilization of outpatient care, using data for 2012.

|  | Cost-sharing effect, JPY | Mean health expenditure at age 69, JPY | Difference in health expenditure, % | Elasticity |
| --- | --- | --- | --- | --- |
| **Panel A: Overall sample** | 1532.5** | 19,719 | 7.8 | -0.12 |
|  | (451.0) |  |  |  |
|  |  |  |  |  |
| **Panel B: Lower income** | 1130.8 | 22,049 | 5.1 | -0.08 |
|  | (1043.4) |  |  |  |
|  |  |  |  |  |
| **Panel C: Middle income** | 1328.4* | 18,899 | 7.0 | -0.11 |
|  | (667.3588) |  |  |  |
|  |  |  |  |  |
| **Panel D: Higher income** | 1981.1** | 19,155 | 10.3 | -0.16 |
|  | (730.4) |  |  |  |

Note: To save space, this table only reports the estimated coefficients for the RD dummy variables (1=age 70 and above, 0=otherwise). Robust standard errors corrected for clustering at the individual level are in parentheses. **: 1%, *: 5%.

# **Table S19.** Effects of the cost-sharing reduction on the utilization of inpatient care, using data for 2012.

|  | Cost-sharing effect, JPY | Mean health expenditure at age 69, JPY | Difference in health expenditure, % | Elasticity |
| --- | --- | --- | --- | --- |
| **Panel A: Overall sample** | 540.2 | 11,646 | 4.6 | -0.07 |
|  | (1031.0) |  |  |  |
|  |  |  |  |  |
| **Panel B: Lower income** | 2709.4 | 14,903 | 18.2 | -0.27 |
|  | (2530.3) |  |  |  |
|  |  |  |  |  |
| **Panel C: Middle income** | -375.2 | 8,824 | -4.3 | 0.06 |
|  | (1446.5) |  |  |  |
|  |  |  |  |  |
| **Panel D: Higher income** | 70.6 | 12,535 | 0.6 | -0.01 |
|  | (1649.9) |  |  |  |

Note: To save space, this table only reports the estimated coefficients for the RD dummy variables (1=age 70 and above, 0=otherwise). Robust standard errors corrected for clustering at the individual level are in parentheses. **: 1%, *: 5%.

# **Table S20.** Effects of the cost-sharing reduction on the utilization of outpatient care, using a generalized linear model.

|  | Coef. | Marginal effect, JPY | Mean health expenditure at age 69, JPY | Elasticity |
| --- | --- | --- | --- | --- |
| **Panel A: Overall sample** | 0.053** | 1087.9** | 19,719 | -0.08 |
|  | (0.012) | (251.3) |  |  |
|  |  |  |  |  |
| **Panel B: Lower income** | 0.011 | 250.3 | 22,049 | -0.02 |
|  | (0.031) | (692.8) |  |  |
|  |  |  |  |  |
| **Panel C: Middle income** | 0.057** | 1125.7** | 18,899 | -0.09 |
|  | (0.018) | (360.5) |  |  |
|  |  |  |  |  |
| **Panel D: Higher income** | 0.074** | 1498.7** | 19,155 | -0.12 |
|  | (0.018) | (363.0) |  |  |

Note: To save space, this table only reports the estimated coefficients for the RD dummy variables (1=age 70 and above, 0=otherwise). We used a generalized linear model (GLM) with a log link and gamma distribution. Robust standard errors corrected for clustering at the individual level are in parentheses. **: 1%, *: 5%.

# **Table S21.** Effects of the cost-sharing reduction on the utilization of inpatient care, using a generalized linear model.

|  | Coef. | Marginal effect, JPY | Mean health expenditure at age 69, JPY | Elasticity |
| --- | --- | --- | --- | --- |
| **Panel A: Overall sample** | 0.066 | 799.2 | 11,646 | -0.10 |
|  | (0.056) | (675.8) |  |  |
|  |  |  |  |  |
| **Panel B: Lower income** | 0.161 | 2535.6 | 14,903 | -0.26 |
|  | (0.100) | (1568.0) |  |  |
|  |  |  |  |  |
| **Panel C: Middle income** | 0.081 | 758.4 | 8,824 | -0.13 |
|  | (0.098) | (910.4) |  |  |
|  |  |  |  |  |
| **Panel D: Higher income** | 0.010 | 126.7 | 12,535 | -0.02 |
|  | (0.087) | (1114.9) |  |  |

Note: To save space, this table only reports the estimated coefficients for the RD dummy variables (1=age 70 and above, 0=otherwise). We used a generalized linear model (GLM) with a log link and gamma distribution. Robust standard errors corrected for clustering at the individual level are in parentheses. **: 1%, *: 5%.

# **Table S22.** Effects of the cost-sharing reduction on the utilization of outpatient care, including two months of data before and after individuals turn 70 years.

|  | Cost-sharing effect, JPY | Mean health expenditure at age 69, JPY | Difference in health expenditure, % | Elasticity |
| --- | --- | --- | --- | --- |
| **Panel A: Overall sample** | 1528.2** | 19,719 | 7.7 | -0.12 |
|  | (234.4) |  |  |  |
|  |  |  |  |  |
| **Panel B: Lower income** | 849.2 | 22,049 | 3.9 | -0.06 |
|  | (658.7) |  |  |  |
|  |  |  |  |  |
| **Panel C: Middle income** | 1493.1** | 18,899 | 7.9 | -0.12 |
|  | (332.0) |  |  |  |
|  |  |  |  |  |
| **Panel D: Higher income** | 1978.6** | 19,155 | 10.3 | -0.16 |
|  | (325.2) |  |  |  |

Note: To save space, this table only reports the estimated coefficients for the RD dummy variables (1=age 70 and above, 0=otherwise). Robust standard errors corrected for clustering at the individual level are in parentheses. **: 1%, *: 5%.

# **Table S23.** Effects of the cost-sharing reduction on the utilization of inpatient care, including two months of data before and after individuals turn 70 years.

|  | Cost-sharing effect, JPY | Mean health expenditure at age 69, JPY | Difference in health expenditure, % | Elasticity |
| --- | --- | --- | --- | --- |
| **Panel A: Overall sample** | 585.0 | 11,646 | 5.0 | -0.08 |
|  | (571.1) |  |  |  |
|  |  |  |  |  |
| **Panel B: Lower income** | 1360.0 | 14,903 | 9.1 | -0.14 |
|  | (1341.7) |  |  |  |
|  |  |  |  |  |
| **Panel C: Middle income** | 810.8 | 8,824 | 9.2 | -0.14 |
|  | (809.6) |  |  |  |
|  |  |  |  |  |
| **Panel D: Higher income** | -106.3 | 12,535 | -0.8 | 0.01 |
|  | (946.1) |  |  |  |

Note: To save space, this table only reports the estimated coefficients for the RD dummy variables (1=age 70 and above, 0=otherwise). Robust standard errors corrected for clustering at the individual level are in parentheses. **: 1%, *: 5%.

# **Table S24.** Effects of the cost-sharing reduction on the utilization of outpatient care, excluding four months of data before and after individuals turn 70 years.

|  | Cost-sharing effect, JPY | Mean health expenditure at age 69, JPY | Difference in health expenditure, % | Elasticity |
| --- | --- | --- | --- | --- |
| **Panel A: Overall sample** | 703.2* | 19,719 | 3.6 | -0.05 |
|  | (305.1) |  |  |  |
|  |  |  |  |  |
| **Panel B: Lower income** | -379.6 | 22,049 | -1.7 | 0.03 |
|  | (890.7) |  |  |  |
|  |  |  |  |  |
| **Panel C: Middle income** | 825.6* | 18,899 | 4.4 | -0.07 |
|  | (415.2) |  |  |  |
|  |  |  |  |  |
| **Panel D: Higher income** | 1248.8** | 19,155 | 6.5 | -0.10 |
|  | (414.9) |  |  |  |

Note: To save space, this table only reports the estimated coefficients for the RD dummy variables (1=age 70 and above, 0=otherwise). Robust standard errors corrected for clustering at the individual level are in parentheses. **: 1%, *: 5%.

# **Table S25.** Effects of the cost-sharing reduction on the utilization of inpatient care, excluding four months of data before and after individuals turn 70 years.

|  | Cost-sharing effect, JPY | Mean health expenditure at age 69, JPY | Difference in health expenditure, % | Elasticity |
| --- | --- | --- | --- | --- |
| **Panel A: Overall sample** | 145.2 | 11,646 | 1.2 | -0.02 |
|  | (705.0) |  |  |  |
|  |  |  |  |  |
| **Panel B: Lower income** | 1397.5 | 14,903 | 9.4 | -0.14 |
|  | (1651.3) |  |  |  |
|  |  |  |  |  |
| **Panel C: Middle income** | 436.7 | 8,824 | 4.9 | -0.07 |
|  | (988.3) |  |  |  |
|  |  |  |  |  |
| **Panel D: Higher income** | -923.4 | 12,535 | -7.4 | 0.11 |
|  | (1177.8) |  |  |  |

Note: To save space, this table only reports the estimated coefficients for the RD dummy variables (1=age 70 and above, 0=otherwise). Robust standard errors corrected for clustering at the individual level are in parentheses. **: 1%, *: 5%.

# **Table S26.** Effect of turning 70 years on income.

|  | Coef. |
| --- | --- |
| **Proportion of lower-income individuals, %** | -0.52 |
|  | (0.79) |
|  |  |
| **Proportion of higher-income individuals, %** | 0.45 |
|  | (0.77) |

Note: To test whether there is a discontinuity in income at age 70, we conducted two types of regression analyses. First, we regressed an indicator for lower-income on the explanatory variables in Eq. (1) except the income category indicators, using a linear age trend. Second, we regressed an indicator for higher-income on the explanatory variables in Eq. (1) except the income category indicators, using a linear age trend. Robust standard errors corrected for clustering at the individual level are in parentheses. **: 1%, *: 5%.

# **Figure S1.** Distribution of outpatient expenditure.


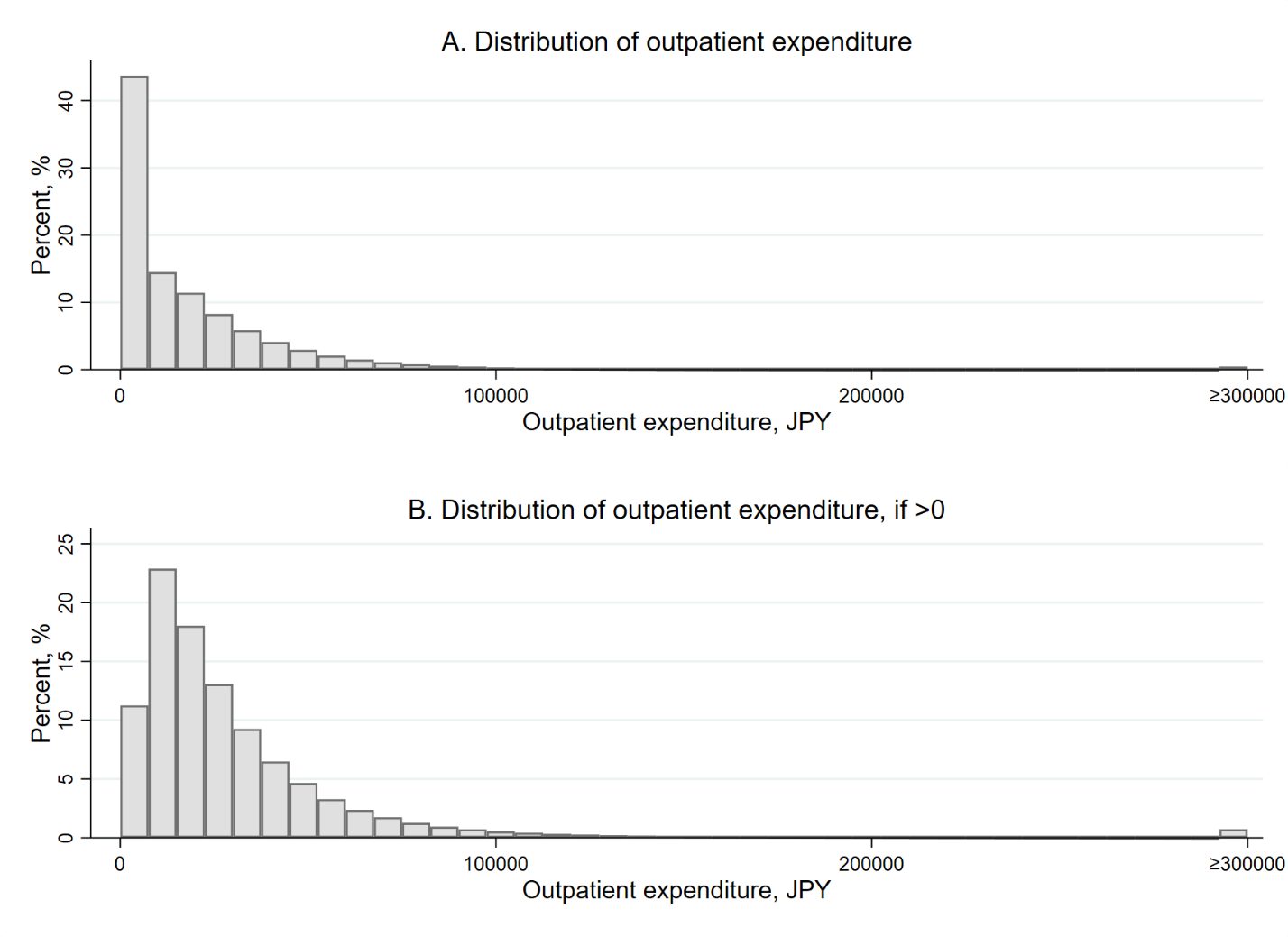


A: Full histogram including zeros. B: Histogram of only the positive values.

# **Figure S2.** Distribution of inpatient expenditure.


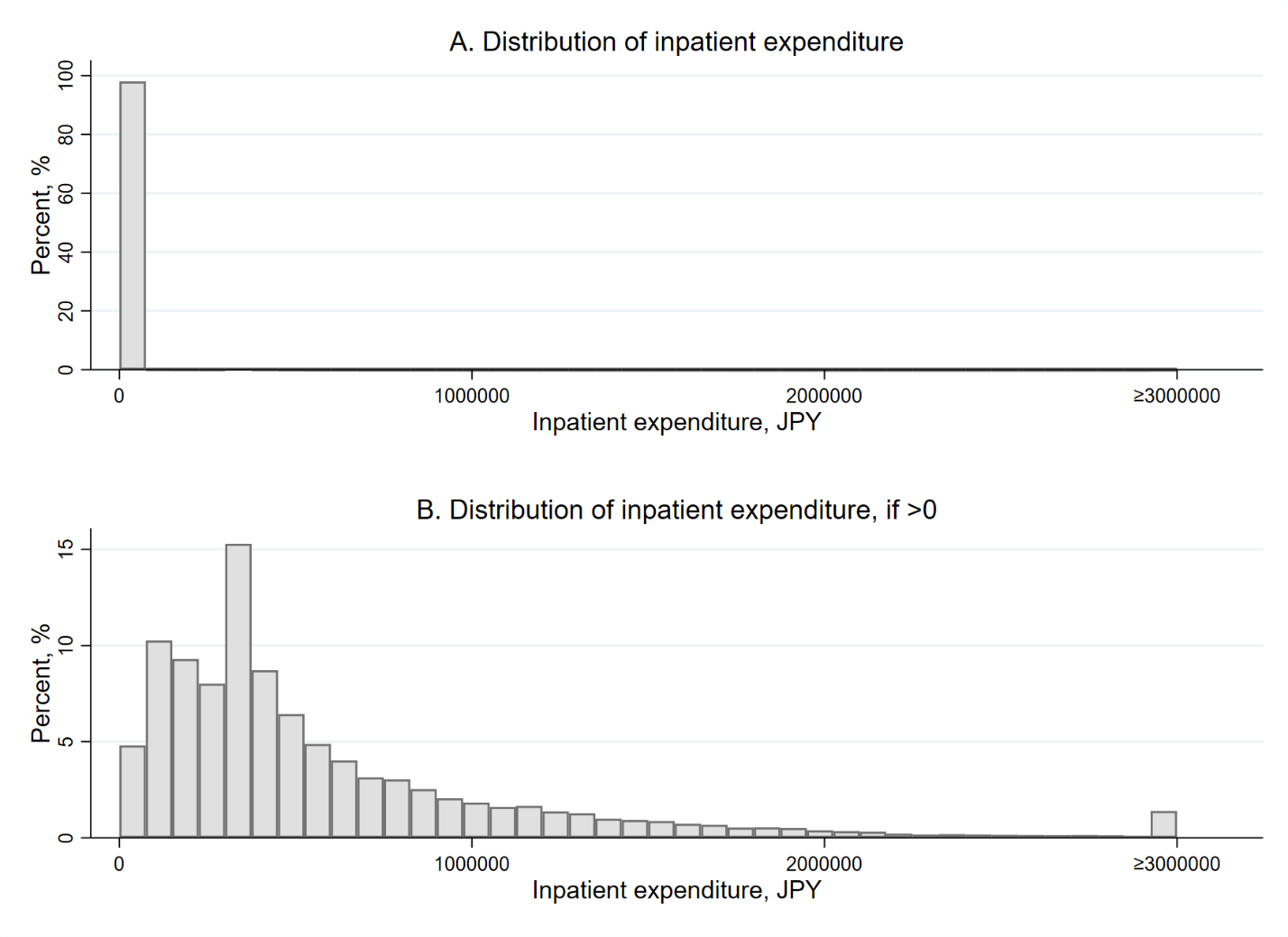


A: Full histogram including zeros. B: Histogram of only the positive values.

# **Figure S3.** Proportions of lower-income or higher-income individuals by age.


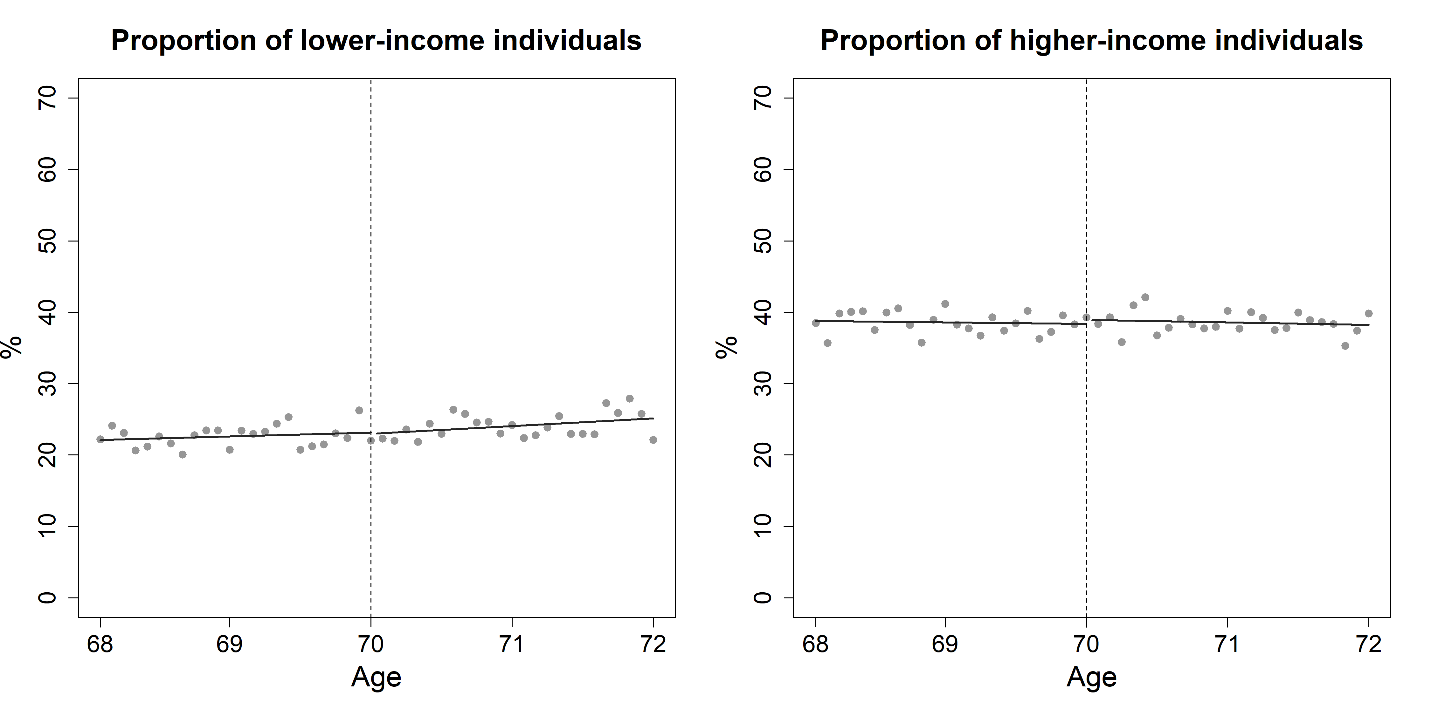


Note: Dots represent the proportion of lower-income or higher-income individuals by age. The vertical dotted lines indicate the age threshold of 70 years. The coinsurance rate was 30% before age 70 and 10% after age 70. Dark lines are from fitting a linear function of age in months, separately for before and after age 70.

# **References**

1. Fukushima, K., et al., *Patient cost sharing and medical expenditures for the Elderly.* Journal of Health Economics, 2016. **45**: p. 115-130.

2. Shigeoka, H., *The Effect of Patient Cost Sharing on Utilization, Health, and Risk Protection.* American Economic Review, 2014. **104**(7): p. 2152-84.

3. Keeler, E.B. and J.E. Rolph, *The demand for episodes of treatment in the health insurance experiment.* Journal of Health Economics, 1988. **7**(4): p. 337-367.
